# An Anatomical Basis for the Myofascial Trigger Points of the Abductor Hallucis Muscle

**DOI:** 10.1155/2020/9240581

**Published:** 2020-01-22

**Authors:** Juliano T. Wada, Flavia Akamatsu, Flavio Hojaij, Ana Itezerote, José Carlos Scarpa, Mauro Andrade, Alfredo Jacomo

**Affiliations:** Department of Surgery-Human Structural Topography-School of Medicine, University of São Paulo, São Paulo 01246-903, Brazil

## Abstract

Myofascial pain syndrome is characterized by pain and a limited range of joint motion caused by muscle contracture related to motor-end-plate dysfunction and the presence of myofascial trigger points (MTrPs). It is the most frequent cause of musculoskeletal pain, with a worldwide prevalence varying between 13.7% and 47%. Of the patients with myofascial pain syndrome, approximately 17% have pain in the medial hindfoot area. The abductor hallucis muscle is located in the medial, posterior region of the foot and is related to painful plantar syndromes. The objective of this study was to describe the distribution of the medial plantar nerve and their anatomical relationship with MTrPs found in the literature. Thirty abductor hallucis muscles were dissected from 15 human cadavers (8 males and 7 females). The muscles were measured, and the distribution data of the medial plantar nerve branches in each quadrant were recorded. For statistical analysis, we used generalized estimation equations with a Poisson distribution and a log logarithm function followed by Bonferroni multiple comparisons of the means. The data are expressed as the mean ± standard deviation. The level of significance was adjusted to 5% (*p* < 0.05). A high concentration of nerve branches was observed in the first quadrant (Q1) of the abductor hallucis muscle, which is the same area in which the MTrPs are described. The topography of the entry points of the branches of the medial plantar nerve to the abductor hallucis muscle correlates with the topography of the muscular trigger points. The anatomical structure of the MTrPs may be useful for a better understanding of the pathophysiology of myofascial disorders and provide a basis for surgical and clinical treatments.

## 1. Introduction

Chronic myofascial pain is the most frequent cause of skeletal muscle pain with prevalence in the world population varying between 13.7% and 47% [1, 2]. Pain is a global public health problem because it directly impacts the individual, the family, and the workplace as it results in a decrease in functional activity [3, 4]. Myofascial pain syndrome may be associated with myofascial trigger points (MTrPs) [5, 6]. MTrPs are foci of muscles that have intense sensitivity and irritability, are located predominantly near the motor end plates, and have palpable tensile band characteristics mediated by the local response of reflex muscle contraction upon palpation of muscle fibers. Increased local irritability gives rise to pain and sensory changes that may be local or referred [7, 8]. The integrated MTrPs hypothesis postulates that, in myofascial pain, the motor endplates release excessive acetylcholine which is evidenced histopathologically by the presence of sarcomere shortening [9].

The abductor hallucis muscle has been gaining prominence in the most recent publications on plantar pain [10, 11]. It is a superficial, triangular, flat, bipenniform muscle that fills the foot medial arch and is responsible for sustaining the arch and hallux abduction and flexion [12, 13]. The muscle originates from flexor retinaculum, medial process of the calcaneus tuberosity, and intermuscular septum between itself and flexor hallucis brevis; the insertion is into the medial aspect of the proximal hallux phalanx, the collateral ligament of the proximal metatarsophalangeal joint, and the medial sesamoid bone [14, 15]. The abductor hallucis muscle is innervated by a medial plantar nerve, which is a branch of the tibial nerve, and the vascular supply is made by a medial malleolar network, medial calcaneus branches of the lateral plantar artery, medial plantar artery, first plantar metatarsal artery, and perforations of the arterial plantar arch [16, 17].

**Figure 1:**
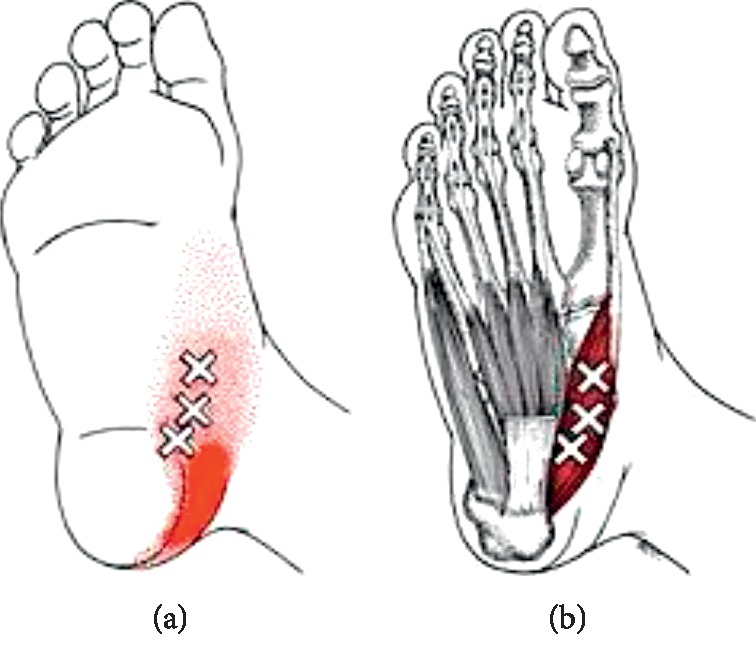
Medial view of the feet, trigger points, and referred pain of the abductor hallucis muscle from Simons et al. [18].

According to Simons et al. [18], there are three areas in the abductor hallucis muscle that have MTrPs. The areas are concentrated in the region of the medial hindfoot and referred pain spreads throughout the medial aspect of the foot (Figure 1). The main epidemiological studies found that 17.4% had foot pains [19], most of them in the region of the medial hindfoot [20, 21].

The authors have sought anatomical approaches to support the theory of myofascial pain syndromes (MPSs) citing the presence of muscle trigger points and providing a pathophysiological explanation [6, 18]. However, some recent researchers refute this approach, concluding that there is no concrete and well-founded pathophysiological explanation for MPS, since, although the muscles have trigger points, patients often do not present with symptoms or some type of electromyographic muscle alteration [22–24].

Another important finding described in a recent study demonstrated that there may be a relationship between the entry points of the nerves to the muscle belly and the myofascial trigger points [25, 26]. A detailed description of the anatomy remains one of the best ways to better understand the pathophysiology and clinical applicability of myofascial trigger points. Nevertheless, the lack of detailed anatomical information still constitutes a major setback for a complete understanding of the physiopathology and the larger clinical applicability of MTrPs.

To better understand MTrPs pathophysiology and to provide more anatomical data for the treatment application based on the hypothesis that trigger points can be related to the muscle innervation, the objective of this study was to observe the anatomical correlation between the clinically described MTrPs and the entry point of the branches of the medial plantar nerve into the abductor hallucis.

## 2. Methods

This study was approved by the Ethics Committee of the Medical School for the Analysis of Research Projects Protocol No. 027/16. To study the interrelationship between the number of entry points of medial plantar nerve branches to the abductor hallucis muscle and the MTrPs of the muscle, human adult cadavers (8 males and 7 females) prepared with 4% phenolic acid and 0.5% formaldehyde were dissected. The cadavers were obtained from a body donation program managed by the Discipline of Human Structural Topography of the Department of Surgery of the University, Medical School. The criteria of the 15 cadavers and 30 feet dissected are as follows: preserved anatomical structures; absence of dissection or previous surgical incision in the feet; age between 40 and 80 years; BMI between 20 kg/m^2^ and 25 kg/m^2^; absence of signs of previous surgeries in the regions of interest; and absence of severe pathologies, anatomical deformities, and processes of necroses, hemorrhages, and fibroses that make adequate study of the tissues of the lower limbs impossible (Table 1).

**Figure 2:**
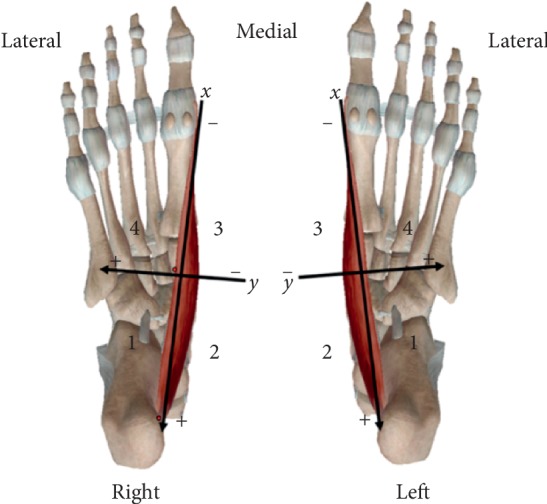
The axes and quadrants of the plantar surface of the feet. 1° quadrant (1Q): posterolateral; 2° quadrant (2Q): posteromedial; 3° quadrant (3Q): anteromedial; 4° quadrant (4Q): anterolateral.

The feet selected met all the study criteria; dissection was performed stratigraphically. Flaps of skin and subcutaneous tissue were folded to expose the abductor muscle of the hallux. The tendons of the long flexors of the digitorum and hallucis longus as well as the tibial artery and vein were separated, and in some cases removed, to identify and isolate the tibial, medial, and lateral plantar nerve. The abductor hallucis muscle was dissected and folded, and the deep surface was carefully dissected to preserve the nerve branches. All entry points of the branches of the medial plantar nerve on the lateral side of the muscle and the medial face of the muscle were demarcated with pins. The boundaries of the quadrants [26, 27] were traced with surgical wires, and the anatomical references were as follows: the *X* axis (anteroposterior) began at the origin (medial tuberosity of the calcaneus) and ended at the insertion (base of the first proximal phalanx), and the *Y* axis (transversal) originated from the midpoint of the *X* axis, close to the navicular bone (Figure 2). The number of entry points of each of the branches of the medial plantar nerve into the muscle in each quadrant was demarcated, and the statistical analysis was performed by a blind evaluator to avoid the observer bias. All the anatomical dissection steps were documented photographically by means of a Nikon D52 camera (Nikon Corporation; Tokyo Japan).

Statistical analysis was performed using SPSS (version 22, IBM). The sample size calculation was based on the results of the first 10 feet evaluated by the pilot project; with this sample, the difference between quadrants 1 and 2 (Q1 and Q2, respectively) was 1.26 points on average with a variability of 1.5 points. The sample used results in a power of 85% and an alpha value of 5%, suggesting a sample size of 26 feet total.

For the quadrant analysis, generalized estimation equations with the Poisson distribution and log logarithm function followed by Bonferroni multiple comparisons were used to evaluate the number of entry points of the medial plantar nerve into the hallucis abductor muscle in the muscle's respective quadrants. Data are presented as the mean ± standard deviation, and the level of significance was adjusted to 5% (*p* < 0.05) for all tests.

## 3. Results

**Figure 3:**
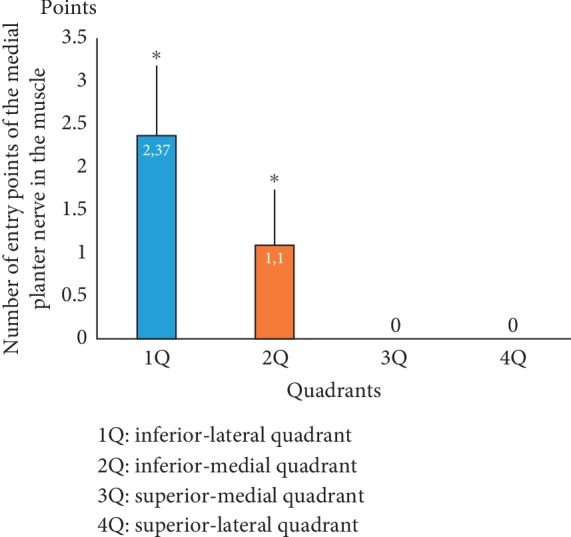
Number of entry points of the medial plantar nerve into the abductor hallucis muscle belly. 1° quadrant (1Q): posterolateral; 2° quadrant (2Q): posteromedial; 3° quadrant (3Q): anteromedial; 4° quadrant (4Q): anterolateral.

**Figure 4:**
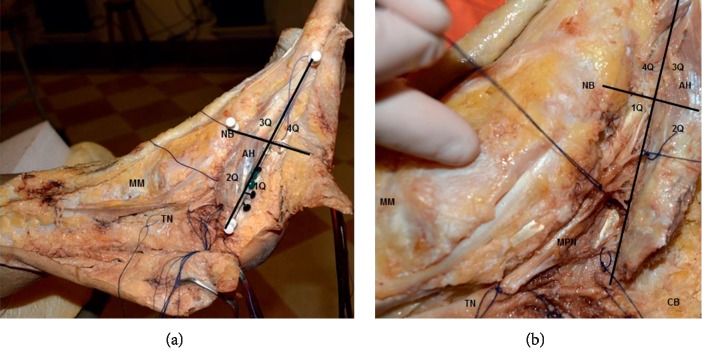
Medial view of the left foot (A and B) and medial plantar nerve branch penetration in the posterolateral region muscle belly. The abductor hallucis muscle was retracted to expose its inner side to view the muscle innervation.

Only one foot (*n* = 1) was excluded from the study because of an orthopedic surgery with intense tissue fibrosis in the ankle that prevented adequate anatomical evaluation; therefore, 29 feet were used for the analysis. The anatomical analysis showed that all of the entries of the branches of the medial plantar nerve were concentrated in the posterior foot area near the calcaneus bone and that both the third and fourth quadrants (3Q and 4Q, respectively) did not contain any entry points of the branches of the medial plantar nerve into the muscle belly. In contrast, the first quadrant (1Q, posterolateral) presented a statistically significant higher number of entry points of the branches of the medial plantar nerve relative to the second quadrant (2Q, posteromedial) (2.37 ± 1.84 points versus 1.11 ± 1.16 points) (*p* < 0.05) (Figure 3). After dissection, it was possible to observe that the medial plantar nerve penetrates the lateral side of the abductor hallucis muscle and crosses the entire second quadrant (medial, 2Q) before innervating the posterolateral (first quadrant, 1Q) region of the muscle. The nerve divides on average into 3 branches to penetrate the muscle in the middle of the muscle belly; briefly, the muscle receives all its innervation in only 1/4 of its total muscle area (Figure 4).

## 4. Discussion

The relationship between the entry points of nerves into the muscle belly and the myofascial trigger points has already been reported by Akamatsu et al. [25, 26]. Nerves are responsible for muscle contraction through the release of acetylcholine from the motor endplate. All the mechanisms involved in the nodular tense band in the muscular belly (i.e., MTrPs) are not completely elucidated, and motor plate disorders caused by an increase of acetylcholine may be one of the causes [28]. Electromyography (EMG) studies have demonstrated that acute and chronic muscle pain experience alters the distribution of activity of the muscle and may cause a shift of the peak muscle activity [29, 30]. An active MTrP causes a clinical pain complaint [18], and the International Association for the Study of Pain [31] refers MTrPs related to many pain conditions and the plantar pain is mainly caused by muscular dysfunction [32]. In this study, it was demonstrated through anatomical dissections that the branches of the medial plantar nerve penetrate the abductor hallucis muscle mainly in the first quadrant (Q1, posterolateral). The first quadrant (1Q, posterolateral) in both groups presented a significant difference relative to the other plantar areas.

The anatomical entry points of the medial plantar nerve into the abductor hallucis muscle corresponded to the described areas of the myofascial trigger points (MTrPs).

In addition, this region of the medial hindfoot is the same as described by Simon et al. [18] and Saggini et al. [33] for the trigger point of the hallucis muscle. The nerve divides on average into 3 branches before penetrating the muscle in the middle of the muscle belly, a similar result as Saitou et al. [34]. The main epidemiological studies found that 17.4% had foot pains [19], most of them in the region of the medial hindfoot [20, 21].

Several studies have examined the pathway of the medial plantar nerve but only up to the margin of the abductor hallucis muscle [35, 36]. The studies that more clearly describe the plantar nerves are from Delfaut et al. [37] and Alshami et al. [35], but they used magnetic resonance imaging only, which fails to demonstrate in detail the innervation of the muscle. Saitou et al. [34] and Minetto et al. [38] demonstrated that the motor endplate of the abductor muscle of the hallux is located mainly in the middle of the muscle and in the posterior region near the muscle origin. We found an anatomical match between the abductor hallucis muscle trigger points area and the anatomical entries of the medial plantar nerve branches into the muscle belly. According to the data described in the present study, it is believed that the muscle region with high nerve concentration (first quadrant, 1Q) may be related to the pathophysiology of myofascial syndromes.

The present study was the first to relate the entry points of the medial plantar nerve into the belly of the abductor hallucis muscle with the myofascial trigger points and to evaluate the standard location of the nerves using the quadrant approach. The location of the trigger points in relation to the entry points of the medial plantar nerve branches into the abductor muscle of the hallux is a logical explanation for understanding the dysfunction of the neurological activity of the MTrPs.

## 5. Conclusion

The branches of the medial plantar nerve penetrate mostly into the first muscle quadrant (1Q, posterolateral) of the abductor muscle of the hallux and represent a strong relationship between nerve anatomy and the location of the myofascial trigger points.

## Figures and Tables

**Table 1:** Baseline sample characteristics.

Anthropometric data	Anatomic dissection group
Gender (M/F)	8/7
Age (years)	60.8 ± 10.7
Height (m)	1.68 ± 0.7
Weight (kg)	69.1 ± 11.6
BMI (kg/m^2^)	24.2 ± 4.3

*Note.* Data are presented as mean ± standard deviation. M/F: male, female; BMI: body mass index; m: meters; kg: kilograms.

## Data Availability

The data used to support the findings of this study are included within the article, but if it is necessary to consult all the data of the table with each of the measurements of the entrance of nerves per quadrant of the corpses feet, they are available from the corresponding author upon request.

## Abbreviations

TN: Tibial nerve
MPN: Medial plantar nerve
MM: Medial malleolus
CB: Calcaneus bone
NB: Navicular bone
AH: Abductor hallucis
1Q: First quadrant
2Q: Second quadrant
3Q: Third quadrant
4Q: Fourth quadrant
